# EU-UK criminal justice and security cooperation after Brexit: A perspective

**DOI:** 10.1016/j.fsisyn.2021.100144

**Published:** 2021-03-09

**Authors:** Tim J. Wilson

**Affiliations:** Criminal Justice Policy, Centre for Evidence and Criminal Justice Studies, Northumbria University Law School, City Campus 1, Newcastle upon Tyne, NE1 8ST, UK

## Abstract

The prospect of significantly reduced and potentially unstable EU-UK criminal justice cooperation under the 2020 Trade and Cooperation Agreement (TCA) unless criminal justice professionals and academics can help to shape its future development.Key wordsPost-Brexit readjustment process; The EU-UK Trade and Cooperation Agreement (TCA); EU-UK criminal justice cooperation

## 1 Continued cooperation, but with reduced scope, efficiency and influence

… an operational downgrade for law enforcement authorities across the UK … compared to what we had before, …though, if you look at it from the parameters of any other third country, we have done rather well …. 1

Part Three of the EU-UK Trade and Cooperation Agreement (TCA)^2^ – the section dealing with criminal justice and security^3^ - indicates a spectrum of continuity/discontinuity in cooperation from January 1, 2021 onwards.^4^ This reveals both an initial (at least) loss of operational efficiency and a diminution of formal British government influence over the strategic development of EU criminal justice law, institutions and operational priorities, for example:

• General continuity: data sharing (biometric and vehicle data via the Prüm arrangements and criminal records), PNR screening and confiscation measures.
• Reduced continuity: the model of extradition based on the European Arrest Warrant (EAW), though at the time of writing the practical significant of some detailed changes are unclear.
• Significant loss of continuity: mutual legal assistance in criminal investigations and evidence gathering, participation in Europol, Eurojust and joint investigation teams.
• Significant discontinuity: a loss of the fully automated and integrated flow of information from 29 countries^5^ into UK police and border control systems via SIS II. EU member states will now need to duplicate all SIS notices onto Interpol communication systems. Whatever arrives in the UK by this route will then need to be manually uploaded before it becomes accessible for policing the streets or controlling entry at the border.^6^
• Almost complete discontinuity: mutual enforcement of financial penalties except in relation to VAT and welfare benefits.

Such a legal analysis, however, may not always correspond to operational performance and professional influence. For example, the time limits for EU-UK criminal record exchange have doubled under the TCA to 20 days. UK law enforcement organisations expect little variation from the average pre-2021 criminal record transmission time of six days because organisational and technical systems will barely change.^7^ Also, they know the value of professional influence within EU institutions.

## 2 Potential instability

Successive UK governments struggled to achieve a post-Brexit relationship that might limit the economic and constitutional damage but would appear to be consistent with Brexit rhetoric. Continued EU-UK criminal justice and security cooperation had also been a priority in their negotiations with the EU, particularly access to SIS II operational information and the EAW extradition model. This presented additional difficulties. This was chiefly because of the misrepresentation of the jurisprudence of the Court of Justice of the European Union (CJEU) and misplaced hostility to international human rights law encouraged earlier by some members those governments.^8^

Up to January 31, 2020 the UK was a member state bound by EU internal treaties. By the end of the year, it had accepted the status of a third country whose relationship with EU law and institutions is regulated (provisionally at the time of writing^9^) by two international treaties. The readjustment of the EU-UK relationship, however, is far from over. The second treaty, the 1,246-page TCA finalised on 24 December provides an overarching politico-legal framework within which the next and probably subsequent chapters of Brexit readjustment will be played out. Its authors and signatories cannot know (even guess with confidence) when and how the readjustment process will end.

The last-minute finalisation of the TCA, whether by accident or design, enabled the UK Government to avoid considered scrutiny prior to the Westminster Parliament’s approval of the Treaty.^10^ During the next phase of Brexit readjustment, however, the UK Government will have less control or influence over how events unfold. The Commission’s unilateral competency over matters of huge economic importance to the UK - regulatory equivalences for financial services, data protection adequacy and compliance with EU sanitary and phytosanitary regimes – together with and concerns over Northern Ireland/British mainland trade and the far from resolved fisheries questions will undoubtedly give rise periodically to political turmoil that could threaten the TCA’s durability. It only requires nine months’ notice for the EU or the UK to withdraw unliterally from any aspect of the Treaty or entirely.

Criminal justice and security cooperation is potentially even more unstable. If the UK is not granted a Commission data adequacy determination or if such a determination is withdrawn, all data sharing - under the TCA or other arrangements (e.g., Council of Europe Conventions and bi-lateral treaties) between the UK and EU member states would be imperilled. In addition, Part Three cooperation may also be terminated should the UK denounce the ECHR or certain of its protocols (chiefly those renouncing the use of capital punishment). These two risks are interwoven under EU law. Data adequacy requires a holistic analysis of rule of law compliance, including constitutional and human rights safeguards. Also, as an associated state, UK national security agencies’ compliance with data protection principles and human rights now comes within the data adequacy scrutiny process.

## 3 Scope for professional and academic engagement to improve efficiency and perhaps reduce instability

The TCA’s initial limitations and uncertain future means that it is essential for criminal justice professionals, including forensic scientists and technologists, to help facilitate the successful implementation and sequential improvement (envisaged in the Treaty) of Part Three cooperation. Academics also have a role in explaining the value of such cooperation for justice; and in challenging erroneous political views about human rights law and the CJEU. In other words, there are important roles for *Forensic Science International: Synergy*’s authors and readers.

Detailed rule-making under the TCA needs to be sufficiently dynamic to respond rapidly to fast changing criminal activity, especially in cyberspace, by quickly permitting the coordinated exploitation of new scientific and technological techniques to counter such threats. In addition, it must either be sufficiently flexible to bridge differences in approaches to law or scientific and technological standards or, better still, encourage anticipative EU-UK expert collaboration to minimise divergence. Fortunately, its governance structure creates opportunities for law enforcement officers, prosecutors, forensic scientists and technologists, even for human rights lawyers and academics to engage with Part Three rule-making and to influence future cooperation strategy:

• In addition to having a key role in dispute resolution, the Specialised Committee on Law Enforcement and Judicial Cooperation (established under the TCA) and its working groups will oversee the implantation and future improvement of Part Three cooperation. This type of committee and working group structure and way of working will already be very familiar to criminal justice professionals who have been involved the implementation of EU legislation. The Committee has specific powers and tasks, such as the ability to amend deadlines for the scientific and technical evaluations required as the UK widens its participation in Prüm data sharing. New responsibilities can be anticipated as EU criminal and mutual security law evolve, scientific standards governing biometric data sharing improve, and Europol and Eurojust mandates change.
• The creation of an EU-UK Parliamentary Partnership Assembly is optional under the TCA, but transparency of and accountability will be enhanced by mandatory consultation with a joint Civil Society Forum about the implementation of the TCA and its extension or revision in supplementary agreements. This again should offer considerable scope for professional and academic participation and influence.

The UK will also continue to participate, as an associate member of the European Research Council in its €95.5 billion (2021–2027) Horizon Europe research programme and Marie Skodowska-Curie Actions.^11^ This will provide opportunities for individuals and institutions to join with EU colleagues in initiatives that will directly or indirectly support Part Three implementation and development.

## Funding

The author received financial support for the research, authorship, and publication of this article from the Arts and Humanities Research Council for funding of the UK-Irish Criminal Justice Cooperation Network (AH/S002197/1).

## Declaration of competing interest

The authors declare that they have no known competing financial interests or personal relationships that could have appeared to influence the work reported in this paper.

## Footnotes

1 N. Ni Loideáin, Uncorrected oral evidence to the House of Lords European Union Security and Justice Sub-Committee: Post-Brexit UK-EU security cooperation Tuesday 12 January 2021, Q11. < https://committees.parliament.uk/oralevidence/1536/pdf/> (accessed 8 February 2021).

2 Multi-lingual text available at < https://ec.europa.eu/info/relations-united-kingdom/eu-uk-trade-and-cooperation-agreement_en >, (accessed 22 January 2021). For important additional texts finalised on 24 December see: n. 11.

3 Part Four is also relevant because it provides for coordinated EU-UK responses to cyber-security threats and cybercrime.

4 Given effect in UK law on 31 December 2020 by the EU (Future Relationship) Act 2020 s.40. < https://www.legislation.gov.uk/ukpga/2020/29/contents > (accessed 12 February 2021).

7 Uncorrected oral evidence on behalf of the National Crime Agency and the National Police Chiefs’ Council to the House of Lords European Union Security and Justice Sub-Committee: Post-Brexit UK-EU security cooperation Tuesday 26 January 2021, Q31. < https://committees.parliament.uk/oralevidence/1606/pdf/> (accessed 8 February 2021).

8 ‘Misrepresentation’ and ‘misplaced’ because of a failure to acknowledge the margin of discretion/appreciation and judicial deference, see: T. J. Wilson, Prisoner Transfer within the Irish-UK Common Travel Area (CTA) after Brexit: human rights between politics and penal reform, *Journal of Criminal Law* (online first) (2020) 1–23. https://doi.org/10.1177/0022018320977527.

9 The EU and UK agreed to apply the TCA provisionally prior to ratification by the European Parliament.

10 Under the EU (Future Relationship) Act 2020 (see n. 4).

11 This could only be agreed in principle on 24 December 2020. See: see *Joint Declaration on Participation in Union Programmes and Access to Programme Services* < https://assets.publishing.service.gov.uk/government/uploads/system/uploads/attachment_data/file/948105/EU-UK_Declarations_24.12.2020.pdf > (accessed 13 February 2021).

## Footnotes

5 All EU member states except Ireland, together with Iceland, Liechtenstein, Norway and Switzerland.

6 Uncorrected oral evidence on behalf of the National Crime Agency and the National Police Chiefs’ Council to the House of Lords European Union Security and Justice Sub-Committee: Post-Brexit UK-EU security cooperation Tuesday 26 January 2021, Q24-Q31 < https://committees.parliament.uk/oralevidence/1606/pdf/> (accessed 8 February 2021).

